# Comparison of Microencapsulation by Emulsion-Solvent Extraction/Evaporation Technique Using Derivatives Cellulose and Acrylate-Methacrylate Copolymer as Carriers

**Published:** 2012-10-07

**Authors:** Mitra Jelvehgari, Seyed Hassan Montazam

**Affiliations:** 1 School of Pharmacy, Tabriz University of Medical Sciences, Tabriz, IR Iran; 2 Drug Applied Research Centre and Faculty of Pharmacy, Tabriz University of Medical Sciences, Tabriz, IR Iran; 3 Islamic Azad University of Bonab Unit, Bonab, IR Iran

**Keywords:** Drug Componding, Copolymer, Drug Carriers

## Abstract

**Background:**

Microencapsulation is a useful method to prolong a drug release from dosage forms and to reduce its adverse effect (1) among various available methods. The microencapsulation of hydrophilic active ingredients requires the use of a polar dispersing phase such as a mineral oil. Acetone/paraffin systems are conventionally used.

**Objectives:**

The current study aimed to investigate two different microencapsulation techniques comparatively, water in oil in oil (w/o/o) and oil in oil (o/o), for theophylline (TH) loaded ethylcellulose (EC), cellulose acetate butyrate (CAB), Eudragit RS and RL microspheres with regard to loading efficiency, release and degradation kinetics.

**Materials and Methods:**

Microspheres were prepared by the emulsification method by solvent diffusion/evaporation technique and different polymers which were incorporated into microspheres to control the release rate of drug. Theophylline (TH) was chosen as a model drug. The emulsion technique was investigated for to prepare theophylline microparticles. EC and CAB and acrylatemethacrylate copolymer corresponding to the above ratios were selected as microparticles wall materials. The effects of type polymers on the physical characteristics and dissolution of the microparticles were also studied. However, the TH loading efficiency (for w/o/o emulsion about 90.64% and o/o emulsion about 73.90/5 to 95.90%) and the TH release kinetics were influenced by the microencapsulation technique.

**Results:**

The results demonstrated that the o/o microspheres (containing of CAB) was most appropriate, providing a high encapsulation efficiency (95.90%) and low initial burst release (6.45%). The microspheres prepared with CAB polymer showed faster dissolution rate than other polymers with 0.75: 1 drug to polymer ratio. The double emulsion technique with EC as wall material gave the high dissolution efficiency (80.48%) of microcapsules.

**Conclusions:**

Eudragit RS microspheres showed higher yield (90%). The release of TH from CAB and Eudragit RL walled microcapsules was slow whilst the release from those of EC and Eudragit RS were faster. The type of polymer and the drug to polymer ratio were found to be the key factors affecting the release profile which could lead to microspheres with desired release behavior.

## 1. Background

Microencapsulation is a useful method to prolong a drug release from dosage forms and to reduce its adverse effect (1) among various available methods. The microencapsulation of hydrophilic active ingredients requires the use of a polar dispersing phase such as a mineral oil. Acetone/paraffin systems are conventionally used. However, incorporation levels of the hydrophilic active ingredient into the microspheres related to the amounts employed in the process are fairly low and, moreover, this system involves a limitation with respect to the types of polymers which may be used, given that it requires the polymer to be soluble in acetone dispersing phase (2). The emulsion/evaporation techniques are traditionally recognized as unsuitable for water soluble drugs and all water soluble substances. Several methods and techniques are potentially useful to prepare polymeric microparticles in the broad field of microencapsulation. The preparation method determines the type and the size of microparticle and influences the interaction ability among the components used in microparticle formulations. Different encapsulation methods result, in most cases, in either a microcapsule or a microsphere. For example, interfacial polymerization and coacervation methods almost always produce a microcapsule, whereas solvent evaporation may result in a microsphere or a microcapsule, depending on the formulation and processing factors. Microencapsulation technique by emulsion solvent removal method has been applied extensively in pharmaceutical industries for various purposes such as controlled drug delivery, masking the taste and odor of drugs, protecting drugs from degradation, and protecting body from the toxic effects of the drugs. Preparing microspheres from w/o or o/o emulsion by solvent evaporation method works best to incorporate a biologically active substance into microspheres. However, it is difficult to remove the large volume of solvents completely from microspheres, and there are other problems related to the safety of the operation and environmental problems. Besides, a mineral or vegetable oil is used as an external oil phase in w/o and o/o emulsion, and hence to collect or wash the resulting microspheres, and the remaining oil in microspheres is a significant problem. Yet further innovative methods have been proposed for the efficient encapsulation of water soluble drugs by the emulsion solvent evaporation technique involving double emulsion (multiple emulsions) formation where an aqueous core material solution is emulsified in a polymer volatile organic solvent solution. The resulting emulsion, which is called the primary emulsion, is emulsified in oil giving a double emulsion of w/o/o type. Extraction of the volatile solvent yields a solid microcapsule with an aqueous core. Since the external phase is an organic solution, there is no problem as mentioned in w/o or o/o method. 

However, the pharmaceutical active ingredient in oil phase does not often dissolve out into the external organic solution so that the incorporation efficiency of the active ingredient into microspheres becomes high. Conventional double emulsion solvent extraction or solvent evaporation methods are limited to solvents that are not too hydrophilic so that the emulsion can be formed and droplets may not stay too long in a liquid state. Cellulose acetate butyrate (CAB) is a cellulose ester with medium butyryl content and low viscosity. It is soluble in a wide range of solvents and compatible with many other resins. Ethylcellulose (EC) is the nonionic, pH insensitive cellulose ether and insoluble in water but soluble in many polar organic solvents. It is used as a non swellable, insoluble component in matrix or coating systems. Researchers like (3-5) have demonstrated the ability of EC to sustain drugs release. Eudragit® RS 100 is a copolymer of ethyl acrylate, methyl methacrylate and a low content of methacrylic acid ester with quaternary ammonium groups. The ammonium groups are present as salts and make the polymers permeable. Eudragit RS100 is of low water permeability and RL100 is of high water permeability (6). They have been used in formulation of oral controlled release dosage forms (7). In the current study, all of them were used as wall materials of microcapsules. In order to understand how drug release is controlled by diffusion through an intact membrane, CAB, EC, Eudragit RS and RL were selected to prepare polymeric membranes under different processing time limits and temperatures (7). An appropriate type and optimum concentration of polymer were used for microencapsulation of a drug by the emulsion solvent diffusion/evaporation technique (7). Theophylline (TH), also known as dimethylxanthine, is a methylxanthine drug used in therapy for respiratory diseases such as COPD (chronic obstructive pulmonary disease) and asthma. The microencapsulation of drugs with CAB has been carried out successfully in either an aqueous or an organic vehicle. CAB polymer exhibits slower rate of in vitro drug release initiated by lag time, which reduces the plasma drug fluctuations, as seen in conventional tablet dosage forms (8). Ethylcellulose and cellulose acetatebutyrate are insoluble polymers. Acrylic derivatives include insoluble polymers with varying degrees of permeability. 

## 2. Objectives

The current study aimed to evaluate microencapsulation by extraction/evaporation technique using the ethylcellulose, cellulose acetatobutyrate and acrylatemethacrylate copolymer (Eudragit RL 100, Eudragit RS 100) within the microparticle formulations.

## 3. Materials and Methods 

Materials and methods were as the ones used in previous studies (8-10). The choice of certain technique that will give rise to an efficient drug encapsulation depends on the hydrophilicity or hydrophobicity of the drug. The microencapsulation method employed emulsion solvent extraction/evaporation, acetone as solvent for cellulose acetobutyrate (CAB) and Eudragit RL, ethanol and methanol mixture as Eudragit RS solvent and acetonitrile and dichloromethane mixture for ethylcellulose (EC) polymers, n-hexane and n-heptane as non-solvent, Sucrose stearate (Crodesta F70), Span 80 (sorbitan monolaurate), Tween 80 (polysorbate 80) as antiaggregating agent. Core materials include TH. TH (1.5 g) and Eudragit RS 100 (3 g) were dissolved completely in a common solvent consisting of acetone and methanol (3:1 ratio) by a magnetic stirrer at 500rpm. The resulting mixture was poured into the 200 ml liquid paraffin with sucrose stearate (3 %w/w) previously cooled to 10 ºC. Then, the resulting emulsion was heated to 35 ºC for 4h (8). The drug suspension (750 mg TH and 1 g CAB in 15 ml acetone) was then emulsified in a 125 ml liquid paraffin/1.5% w/w ester sucrose solution under stirring at 400 rpm (9). 

The drug suspension (2 g TH and 0.5 g Eudragit RL100 in A mixed solvent system consisting of acetone and methanol in a 2:1 ratio) was emulsified in a 70 ml liquid paraffin/ 1% w/w Span 80 solution under stirring at 900 rpm (9). The initial W/O emulsion was prepared by adding 2 ml of water to the drug-polymer solution (300 mg EC and 150 mg TH) while stirring at 500 rpm. This W/O primary emulsion was slowly added to 50 ml of light liquid paraffin containing 0.5% span 80 while stirring at 1000 rpm, immersed in an ice water bath. After 2 h, 10 ml of n-hexane (non-solvent) was added to harden the microspheres (10). 

Release was measured as done previously, from a rotating basket apparatus into water at 37 C or as stated, microparticles contain 200 mg theophylline being the sample weight corresponding to Theophylline SR® (Daru pakhsh, Iran). Unlike most of the conventional formulations, TH formulated by microspheres has a longer half-life, which requires lesser dosing and thus increases the patients` compliance. Microencapsulation of TH as microspheres for oral use has been employed to sustain the drug release and to eliminate the chance of dose dumping. TH incorporated with microspheres formulated as multiparticulate drug delivery systems spread out more uniformly in the gastrointestinal tract. This results improvement of drug absorption and reduces plasma pulsation when compared to single unit dosage form of TH. Hence the therapeutic and patients` compliance increase significantly. 

### 3.1. Commonly Used Polymers

TH has been formulated into microspheres by nonbiodegradable polymers and various methods for oral applications. To be used in controlled drug delivery formulations successfully, a material must be chemically inert. It must also have an appropriate physical structure with minimal undesired aging, must be readily processable, should not invoke an inflammatory or toxic response, must be metabolized in the body after fulfilling its purpose, must leave no trace, must be easily processable into the final product form, and must have acceptable shelf life (11).

### 3.2. Methods of Preparation

Micro particulate drug delivery technology represents one of the frontier areas of pharmaceutical science, which involves multidisciplinary scientific approaches, contributing to human health care (11). The major microencapsulation technique which can be employed to formulate TH incorporated in microspheres is briefly discussed below:

### 3.2.1. Coacervation Method

This method is simple and utilizes an aqueous system for the preparation. This process consists of 3 steps under continuous stirring. The steps are: Formation of three phases, then: Dispersing a core material in a solution of coating polymer, immiscible polymer in liquid state (Coating material phase), and coating is accomplished by controlled physical mixing of coating solution and core material in the liquid manufacturing vehicle phase. Rigidisation could be achieved by thermal, chemical cross-linking or desolvation techniques (11).

### 3.2.2. Emulsion Solvent Evaporation/Extraction Method

**Figure 1:**
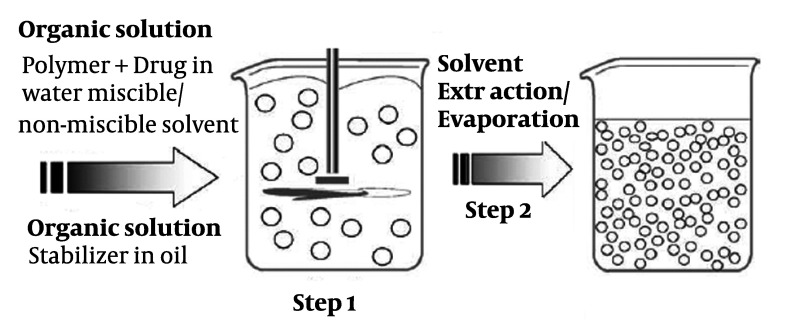
Schematic representation of the single emulsification-extraction/ evaporation technique.

**Figure 2:**
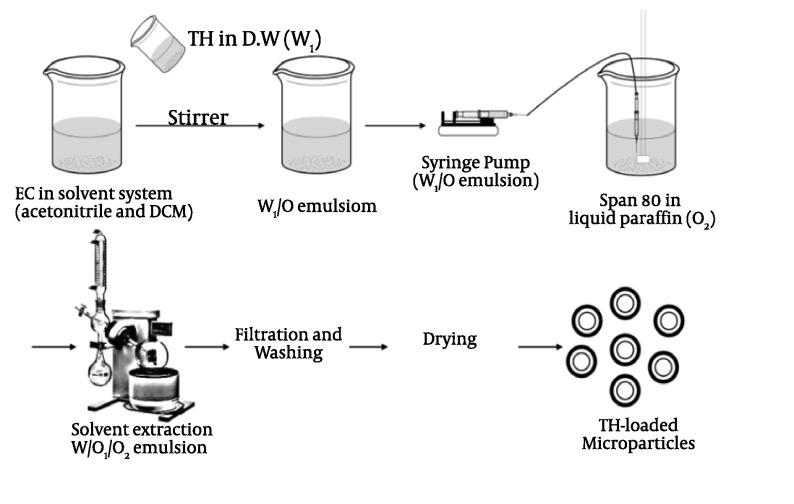
Schematic representation of EC microparticles preparation using double-emulsification the W/O1/O2 method.

In the emulsion solvent evaporation/extraction process, a polymer solution containing drug is emulsified in an immiscible/miscible solvent known as non-solvent, and polymer deposition around the drug particles occurs as a result of partitioning of the polymer solvent from the dispersed phase to the continuous phase (Figures 1 and 2), followed by removal of the polymer solvent through evaporation/extraction (12).

However, the solvent evaporation technique is often not preferred because active ingredient is often lost during the solvent extraction process. This is because the process involves emulsification into an aqueous phase, and a water soluble drug will often rapidly partition from the more hydrophobic polymer solution phase into the aqueous surroundings. Encapsulation by the solvent evaporation process also leads to the production of microspheres. The active ingredient to be encapsulated is traditionally dispersed in a polymer solution of a volatile organic solvent. This phase is emulsified by means of an active surface agent in a non-miscible dispersing medium (water or mineral oil). The organic solvent evaporates by stirring. After the evaporation, the microspheres are recovered by filtration or centrifugation. Solvent evaporation is simple, more flexible and easier to industrialize than other processes such as phase separation or coacervation, and it makes it possible to use reduced amounts of solvent. The microencapsulation of hydrophilic active ingredients requires the use of a polar dispersing phase such as a mineral oil. Acetone/paraffin systems are conventionally used. The components are initially dissolved in a mixture of acetonitrile/ethanol and optionally water (10), or only acetone (9) or in a mixture consisting of methanol and ethanol (8). As used herein, the term “drug phase” refers to the polymer/active agent containing phase formed during microparticles manufacture according to the invention which results from adding an active agent to the organic polymer solution as EC, CAB (8-10) existing prior to the addition of the aqueous surfactant phase. The drug phase may be a solution, dispersion, suspension, or emulsion.

### 3.2.3. Solvent Extraction

This method which is used to prepare microparticles, involves organic phase removal by extraction of the organic solvent. The method involves external phase miscible organic solvent such as mixing acetonitril and dichloromethane; organic phase is removed by extraction with liquid paraffin. The rate of solvent removal by extraction method depends on the temperature of external phase, ratio of emulsion volume to the mineral oil and the solubility profile of the polymer (Figure 1 and 2).

### 3.3. In vitro Drug Release

Drug release on the microspheres was carried out by a USP basket method for 24 h at a stirring speed of 100 rpm and temperature of 37 ± 0.5 °C. An amount of the microspheres equivalent to 200 mg of TH (corresponding to weight of Theophylline SR® from Daru pakhsh, Iran).filled in a hard gelatin capsule (Size no.0) was placed in the dissolution medium containing 900 ml of hydrochloric acid (0.1 M) buffer solution (pH 1.2). After 2 h, 17 ml of 0.2 M phosphate buffer stock, pre-equilibrated at 37 °C, was added to the dissolution vessel. The pH was immediately adjusted, if necessary, with 0.2 N HCl or 0.2 N NaOH to pH 7.4 (19). A quantity (3 ml) of the dissolution medium was sampled at predetermined time intervals and fresh dissolution medium was simultaneously used to replenish the dissolution medium on each occasion to keep the volume constant. The sample was filtered through filter disc (0.45 µm), and the drug concentration in the samples was assayed spectrophotometrically for both the acidic and enteric buffers. Each experiment was repeated three times.

## 4. Results

### 4.1. The Results of Microencapsulation Containing Different Polymers

The hydrophilic substances encapsulated in polymeric microspheres are commonly released following a pattern of three main steps. First, the burst release phase, usually occurring during the first day and mainly determined by the drug in the surface, channels and pores of the microspheres, which were filled by the incubation media for a few hours at the beginning of the trail. Secondly, the slow release phase, releasing few or no drug at all. The third and the last phase comprises a faster release of drug due to the erosion of particles. Occasionally, the release can occur in two steps and the profile shows an asymptotic pattern.

Several processes contribute to the release of the encapsulated drugs, such as diffusion through pores and channels, and exposure of drug molecules to the incubation media, due to the superficial erosion polymeric matrix. The cannels and pores are formed during the assembly of the particles or result from polymeric degradation. Therefore, factors influencing the release profile include the properties of the polymeric matrix, and the drug used in the structure of the microparticle, the encapsulation technique and the experimental conditions, as well as the coencapsulation of additives for several purposes. Emulsification solvent extraction/evaporation involves two steps. The first step requires emulsification of the polymer solution into an organic phase (Figure 1). During the second step polymer solvent is extracted/evaporated, including polymer precipitation of microparticles. A polymer organic solution containing the dissolved drug is dispersed into microparticles, using a dispersing agent; the solvent is subsequently extracted/evaporated by increasing the temperature under pressure or by continuous stirring (13). The size can be controlled by adjusting the stir rate, type and amount of dispersing agent viscosity of organic and aqueous, and temperature (14). The preparatory parameters are summarized in  Table 1. Higher actual drug loading were obtained by increasing the theoretical drug loading. In cases, the encapsulation efficiencies acrylate methacrylate copolymer as Eudragit RL and RS were 73.9-87.21% and the encapsulation efficiencies derivatives cellulose as EC and CAB were similar and greater than 90.64-95.9%. Depending on therapeutic requirements, microspheres with varying drug contents could therefore be prepared through variation of the theoretical drug loading.

**Table 1:** Effect of drug: polymer ratio, stirring rate, dispersing medium and non-solvent on the content, production yield and particle size with different type of polymers in theophylline microparticles

Formulations	Emulsion method	Polymer type	Drug/polymer ratio	Production yield, % ± SD	Theoretical drug content, % ± SD	Mean amount of drug entrapped, % ± SD	Drug loading efficiency, % ± SD	Mean particle size, μm ± SD
F_RS_	O/O	Eudragit RS	5 :1	81.7 ± 3.79	14.29	12.21 ± 0.04	87.21 ± 0.28	260.37 ± 1.69
F_EC_	W/O/O	EC	0.5 : 1	55.24 ± 1.19	33.33	29.53 ± 4.92	90.64 ± 1.32	757.01 ± 2.72
F_CAB_	O/O	CAB	0.75 : 1	45.4 ± 0.45	43	41.10 ± 0.40	95.9 ± 0.95	273.6 ± 1.73
F_RL_	O/O	Eudragit RL	4 : 1	59.1 ± 0.65	80	59.1 ± 0.25	73.9 ± 0.16	372.4 ± 1.70

### 4.2. TH Loading Efficiency Obtained From Single/Double Emulsion

#### 4.2.1. Extraction/Evaporation Technique

The TH entrapment efficiency was calculated as a percentage of drug entrapped ratio in the microspheres to the initial amount of drug added to the system. Results are indicated in Table 1. It was found that the TH entrapment efficiency was rather low (< 73.9 % for Eudragit RL than Eudragit RS with 87.21%). The entrapment efficiency of Eudragit RS100 microspheres was higher than that of the Eudragit RL100 microspheres. This behavior can be explained on the basis of differences of the chemical structures and the % content of quaternary ammonium groups. Eudragit RL100 contains higher amount of quaternary ammonium groups (10%), which facilitates the diffusion of a part of entrapped drug to the surrounding medium during preparation of microspheres. Eudragit RS100 has a thick polymeric surface due to the lower amount of quaternary ammonium groups (5%), which restricts the migration of drug particles to the surrounding medium and also helps to sustain the drug. TH is slightly soluble in water and insoluble in the organic phase in which the EC, Eudragit RS and RL, CAB was dissolved. As a result, TH dispersed in the polymer solution will be extracted by the external phase. However, taken together, the entrapment efficiency of TH using the single emulsion preparation (O/O) was suitable (73.9-95.9%) for practical applications and that TH was found back almost quantitatively (90.64%) in double emulsion preparation with EC polymer. The results suggest that the single emulsion technique (O/O) is suitable for preparation of TH-loaded Eudragit RS, RL and CAB microspheres. Therefore, an alternative method, namely the double emulsion solvent extraction/evaporation method, was investigated to prepare TH-EC microspheres with a high loading efficiency.

### 4.3. Particle Size

The average particle size was determined by laser light scattering particle size analyzer (SALD-2101, Shimadzu, Japan). The microspheres were observed to be 260.37, 757. 273.6 and 372.47µ for Eudragit RS, EC, CAB and Eudragit RL respectively. The particle size of the microspheres obtained by acetonitrile and dichloromethane was much larger than those obtained by methanol and acetone/only acetone. It had been previously reported that using acetone as a co-solvent decreased the particle size (15). In the current study, addition of acetone to methanol also decreased the size of TH-loaded Eudragit RS microspheres (260.37 µ). Acetone is water-miscible while dichloromethane is water-immiscible. Acetone is miscible with methanol as well as dichloromethane. Consequently, the addition of acetone to methanol increases water solubility of the halogenated solvents resulting in an extraction of the solvent by the external phase. Due to the solvent extraction, an interfacial turbulence occurs between the organic polymer phase and the external phase leading to the formation of small particles.

### 4.4. In vitro Release Studies

**Figure 3:**
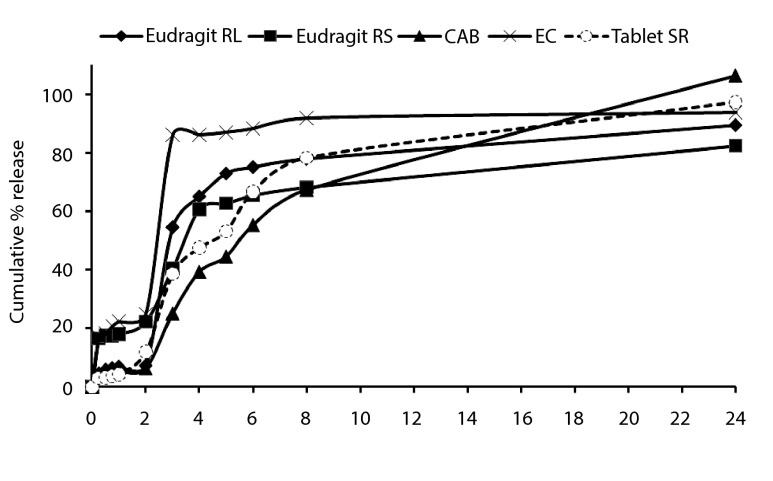
Cumulative percent release of theophylline from microspheres prepared with different type of polymers, and theophylline SR® tablet.

Dissolution rate of polymer coat determines the release rate of drug from the microcapsule when the coat is soluble in the dissolution fluid. Thickness of coat and its solubility in the dissolution fluid influence the release rate. The polymer coat of microcapsule acts as semi-permeable membrane and allows the creation of an osmotic pressure difference between the inside and the outside of the microcapsule and drives drug solution out of the microcapsule through small pores in the coat. The drug release behavior of microsphere formulations and tablet SR (200 mg) are shown in Figure 3, respectively. TH in vitro release from microspheres containing EC, Eudragit RS, CAB and Eudragit RL exhibited initial burst effect which may be due to the presence of some drug particles on the surface of the microspheres. Table 2 shows the dissolution efficiency and difference factor values for microsphere formulations dissolution profiles and tablet SR. Dissolution efficiency and difference factor were used to compare the potential parameters and evaluate the dissolution profiles of different products. Comparison of various dissolution profiles is analyzed by several special measures including the dissolution Rel_2_ (amount of drug release after 2h), Rel_8_ (amount of drug release after 8h), efficiency (DE %) and the difference factor (F_1_) (8). The difference factor is used to determine whether the test product is different to the reference products. An F1 value higher than 0% means that the average difference between both dissolution profiles is less than 15% at all sampling points indicating difference of the two products (16). The DE value for the total time profile of 1440 minute indicated higher dissolution efficiency for the F_EC_ compared to commercial tablet SR and other microspheres. Further, F_1_ (%32.59, %67.91, %19.91 and %30.44) for F_EC_, F_RS_, F_CAB_ and F_RL_, respectively showed difference in the dissolution profiles between their microspheres and tablet SR. The difference between DE values at 1440 minutes was statistically significant (P < 0.05).

Microspheres with high loading efficiency (F_CAB_ and F_RL_ formulations) showed lower dissolution rate for Q2h (6.45% and 7.41%, respectively). Figure 3 and Table 2 indicated that the initial drugs release for some of microsphere formulations were slightly high (F_RS_ and F_EC_). F_CAB_ and F_RL_ formulations showed the lowest burst release in comparison with theophylline SR. The burst release could be attributed to the presence of some TH particles on the surface of microspheres. When particles are prepared by O1/O2 or W/O1/O2 method, Water-soluble drugs do not have the tendency to migrate to the non-polar medium, thereby concentrating on the surface of the microspheres lead to burst effect. Moreover, the burst release could also be explained by the imperfect encapsulation of the drug inside microparticles, resulting from the unstable nature of the emulsion droplets during the solvent removal step. This potential instability may cause a part of the loaded drug to relocate at the microparticle surface, thereby would be rapidly released. Figure 3 also shows that in most cases a biphasic dissolution pattern existed, where pH of the dissolution medium was altered from 1.2 to 7.4. Comparing the drug release from microspheres containing 4 polymers (Figure 3) showed that the release of drug from these microspheres (FCAB and FRL)was slower than that of microspheres containing F_RS_ and F_EC_ (25% and 22.24%, respectively). However, no significant difference was observed between the percentages of drug released at 8h (Q8) microspheres containing F_RL_ and commercial tablet SR (P > 0.05). The first portion of the biphasic dissolution curves is due to TH dissolution which starts immediately after the beginning of the dissolution process. To release the drug in the second phase combination of the diffusion of the remaining dispersed drug into the bulk medium, formation of pores within the matrix due to the initial drug dissolution and swelling which enhances the permeability of the polymer to the drug might be involved (8). Figure 3 illustrates that different TH microspheres exhibited different dissolution profiles. In order to find out which release profiles was more suitable for oral administration, the release data were compared with those of commercial TH extended release formulations. The TH microspheres prepared in this study could be embedded into soft gelatin capsules for peroral administration. According to the US pharmacopoeia not less than 70-80% of the TH should be released within 8 h. The difference factor showed that microsphere formulations containing EC, CAB, Eudragit RL and RS and did not match the release profile of commercial formulations (Table 2) and there was no significant similarity among these dissolution profiles (f1 = 19.91-67.91%). CAB has a low permeability to drug which results from its high intermolecular attraction. Hydrogen bonding between the hydroxyl groups of the carboxylic moiety and the carbonyl oxygen of ester group increases the degree of solidity of the polymer and decreases its porosity and permeability. However, Eudragit RL and RS are a copolymer of acrylic and methacrylic acid esters with a low and high content of quaternary ammonium groups. The ammonium groups present as salts promote permeability and act as a channeling agent for the entrance of the liquid medium through the floating microsphere wall, causing it to swell. Eudragit RL100 microspheres was a little higher than that of Eudragit RS100 microspheres because Eudragit RL100 contained higher amount of quaternary ammonium groups, which rendered it more permeable and accelrated the drug release as reported. These observations could be attributed to the fact that RS100 microspheres have thicker polymeric surface as compared to Eudragit RL100 microspheres. The thick polymeric barrier slows the entry of surrounding dissolution medium in to the microspheres and hence less quantity of drug leaches out from the polymer matrices of the microspheres exhibiting slow release with a lag time of 2 h. However, Eudragit RS 100 microspheres showed a three phase composition. First, an initial release due to the drug desorption from the particle surface; secondly, a lag time for a certain period, resulting from the diffusion of the drug into microsphere surface; and thirdly, a constant sustained release of the drug resulting from the diffusion through the polymer wall as well as its erosion. This facilitates the diffusion of the dissolved drug out of the microsphere into the dissolution medium. Thus, by varying the ratio of CAB, Eudragit RL, and RS in the TH microspheres, TH release rate can be controlled. CAB polymer exhibit slower rate of in vitro drug release initiated by lag time, which reduces the plasma drug fluctuations, as seen in conventional tablet dosage forms (8). Acrylic derivatives include insoluble polymers (EC, CAB) with varying degrees of permeability.

**Table 2:** Comparison of various release characteristics of theophylline from different type of polymers in microparticles formulations, and theophylline SR® Tablet

Formulation	codeQ 2 a, %	Q 8 b, %	DE c	T d 50%, h	f1 e
F_EC_	25 ± 2.10	91.87 ± 3.40	80.48 ± 4.21	4	32.59 ± 2.23
F_RS_	22.24 ± 1.16	68.27 ± 1.22	67.8 ± 3.55	3.5	67.91 ± 4.42
F_CAB_	6.45 ± 0.16	71.39 ± 2.06	69.39 ± 4.01	5	19.91 ± 1.23
F_RL_	7.41 ± 0.03	77.97 ± 1.17	72.36 ± 5.52	> 3	30.44 ± 3.67
Theophylline SR_®_	12.89 ± 1.55	80.86 ± 5.73	73.72 ± 3.98	4	0

^a^Amount of drug release after 2h

^b^Amount of drug release after 8h

^c^Dissolution Efficiency

^d^Dissolution time for 50% fractions

^e^Difference factor

## 5. Discussion

Dissolution efficiency (DE) was calculated from the area under the dissolution curve at time and expressed as percentage of the area of the rectangle described by 100% dissolution at the same time. Microspheres (containing Eudragit RL, RS, and CAB) showed lower dissolution efficiency 67.80 to 72.36% and slow dissolution. Theophylline SR® tablet (73.72%) had higher release in comparison with those of microspheres (P > 0.05), (Table 2 and Figure3). However, the DE values microspheres containing EC (80.48%) showed that they were statistically more significant than commercial tablet (P < 0.05). The in vitro release profiles were fitted on various kinetic models in order to find out the mechanism of drug release (17, 18). The fit parameters to Higuchi, first order, Peppas and zero order equations are given in Table 3. The rate constants were calculated from the slope of the respective plots. High correlation was observed for the Peppas model (for EC microspheres), Higuchi (for CAB, Eudragit RL and RS microspheres) and first order (theophylline SR). The data obtained were also put in Korsemeyer-Peppas model in order to find out n value, which describes the drug release mechanism. The n value of microspheres of different polymers was between 0.21-0.89, indicating that the mechanisms of the drug release were diffusion (for EC and Eudragit RS) and erosion (for CAB and Eudragit RL) controlled. 

TH can be developed successfully as a controlled drug delivery system in the form of EC, CAB, Eudragit RL and RS microspheres. The polymeric microspheres can be prepared by the single/double emulsion technique. These formulations can be a choice of treatment for management of chronic asthma with much more patient comfort without side effects. TH microspheres were prepared successfully by the solvent evaporation/extraction method. Types of Polymers influences the particle size as well as drug release pattern of microsphere. The yield was high and encapsulation efficiency was good for Eudragit RS and CAB microparticles, respectively. The particle size increased, when TH microparticles were prepared by double-emulsion technique (for EC polymer). Initially at gastric medium (pH 1.2), much less release of drug (TH) from microspheres was found, but pH 7.4, all formulations showed burst release initially and then tendency to release at constant rate. Type of polymer and technique of microencapsulation influence the particle size and drug release properties. The results demonstrate that the o/o microspheres (containing of CAB) is most appropriate (95.90%), providing a high encapsulation efficiency and low initial burst release (6.45%). The assessment of release kinetic showed that drug release from TH microspheres followed the Higuchi model with diffusion (Eudragit RS microparticles) and erosion (Eudragit RL and CAB) controlled drug release mechanism. However, EC microcapsules showed Peppas model. 

Unlike most of the conventional formulations, TH formulated with microspheres have a longer half-life, which requires lesser dosing and thus increases the patient compliance. Microencapsulation of TH as microspheres for oral use has been employed to sustain the drug release and to eliminate the chance of dose dumping. TH incorporated with microspheres formulated as multiparticulate drug delivery systems spread out more uniformly in the gastrointestinal tract,which resultsdrug absorption improvement and reduces plasma pulsation when compared to single unit dosage form of TH. Hence the therapeutic and patient compliance increases significantly. 

**Table 3:** Fitting parameters of the in vitro release data to various release kinetics models

	F_EC_	F_RS_	F_CAB_	F_RL_	Theophylline SR
**Zero f=kt**					
K	0.0006	0.0008	0.0428	0.0005	0.0007
RSQ	0.5148	0.9114	0.8421	0.3848	0.6869
D(SS), %	723.1258	701.1646	426.1421	712.4172	459.3552
**First Ln(1-f)=k**					
K	0.0022	0.0031	0.3844	0.0014	0.0032
RSQ	0.7205	0.8517	0.9452	0.5976	0.9936
D(SS), %	309.546	373.0134	2419.9759	525.5641	745.9072
**Peppas** **Lnf=lnk+blnt**					
b	0.34091	0.2076	0.8920	0.7511	1.2330
K	0.0518	0.1894	0.0802	0.0045	0.0004
RSQ	0.9433	0.8757	0.8166	0.5780	0.9725
D(SS), %	22.4856	23.9331	327.5811	270.5196	97.9302
**Higuchi f=kt ^0.5^**					
K	0.0295	0.0381	0.2465	0.0218	0.0339
RSQ	0.7260	0.9344	0.9478	0.7096	0.8721
D(SS), %	201.3116	225.0460	1753.5811	950.3229	1398.717
